# Testicular infarction as a rare complication of pyogenic epididymoorchitis due to *Pseudomonas aeruginosa*: A case report and systematic literature review

**DOI:** 10.1016/j.idcr.2021.e01258

**Published:** 2021-08-25

**Authors:** Kazuhiro Ishikawa, Takahiro Matsuo, Tomoaki Nakamura, Fujimi Kawai, Yuki Uehara, Nobuyoshi Mori

**Affiliations:** a Department of Infectious Diseases, St. Luke’s International Hospital, Tokyo 104-0044, Japan; b Department of Pulmonary Medicine, St. Luke’s International Hospital, Tokyo 104-0044, Japan; c St. Luke’s International University Library, Tokyo 104-0044, Japan; d Department of Clinical Laboratory, St. Luke’s International Hospital, Tokyo 104-0044, Japan; e Department of Microbiology, Juntendo University Faculty of Medicine, Tokyo 113-8431, Japan; f Department of General Medicine, Juntendo University Faculty of Medicine, Tokyo 113-8431, Japan

**Keywords:** Testicular infarction, Pyogenic epididymoorchitis, *Pseudomonas aeruginosa*

## Abstract

**Background:**

Testicular infarction is a known serious complication associated with epididymitis. It is known to be idiopathic in 70% of cases but the frequency, risk factors, and management are yet to be elucidated. This paper aims to report a case of testicular infarction secondary to pyogenic epididymoorchitis caused *by Pseudomonas aeruginosa.*

**Case presentation:**

A 64-year-old male with a past medical history of benign prostate hypertrophy using intermittent self-catheterization and a recent history of culture-negative pyogenic epididymoorchitis treated with oral cefpodoxime was admitted to our hospital due to a 4-week history of fever, right scrotal pain, and swelling. Scrotal ultrasonography showed a hypoechoic testis without testicular torsion. He was diagnosed with testicular infarction and a scrotal abscess due to *Pseudomonas aeruginosa*, and was treated with cefepime along with transcutaneous drainage. Despite the antimicrobial treatment, he experienced testicular loss with necrotic tissue. Because little is known about the risk factors, clinical characteristics, management, and prognosis of testicular infarction secondary to epididymitis, we performed a systematic review of the literature.

**Conclusion:**

This is a case of testicular necrosis during the treatment of epididymitis with negative urine culture and detection of *Pseudomonas aeruginosa* in tissue culture. Clinicians should perform frequent blood flow evaluation to the testis for early urologic intervention.

## Introduction

Testicular infarction is a rare but serious complication associated with epididymitis. Up to 70% of testicular infarctions are reportedly idiopathic [1], and their frequency, risk factors, and management remain unknown. Herein, we present a case report of testicular infarction secondary to pyogenic epididymoorchitis caused *by Pseudomonas aeruginosa*. We also performed a systematic literature review pertaining to testicular infarction associated with epididymitis.

## Case

**Fig. 1:**
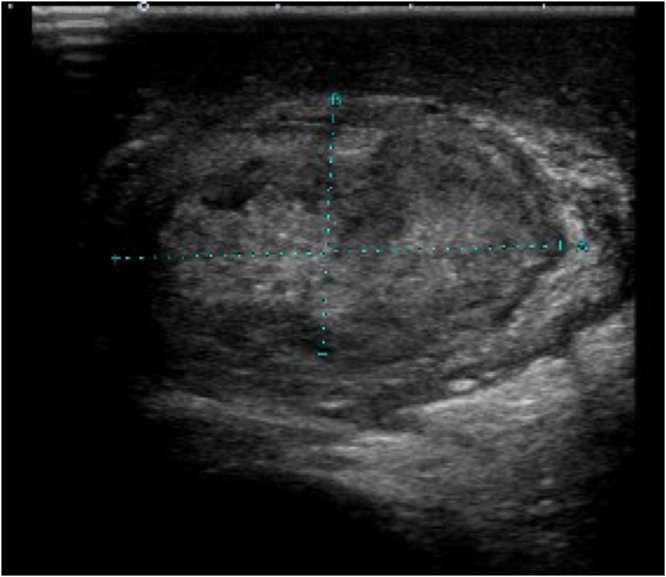
Scrotal ultrasound showing hypoechoic testis without testicular torsion.

**Fig. 2:**
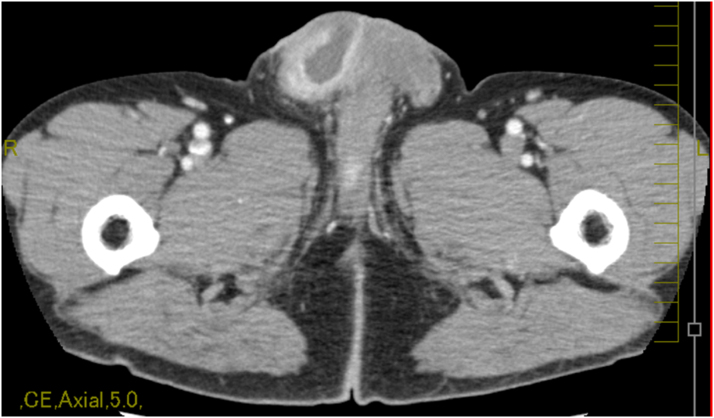
Computed tomography scan showed fluid correction with contrast effect in the capsule of right scrotum.

**Fig. 3:**
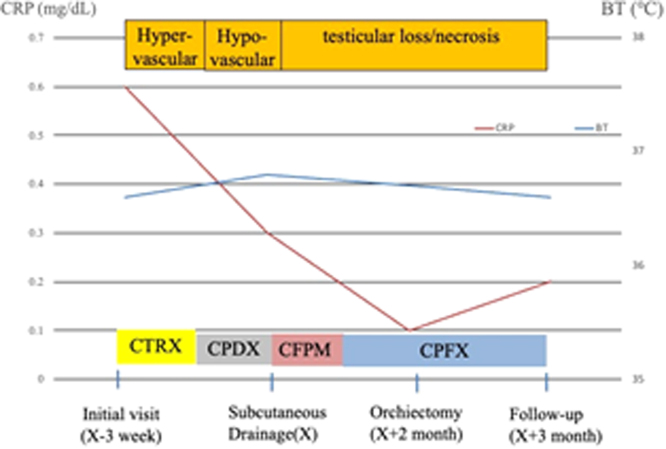
Clinical course. CTRX: ceftriaxone, CPDX: cefpodoxime, CFPM: cefepime, CPFX: ciprofloxacin, CRP: C-reactive protein, BT: body temperature.

A 64-year-old male with a past medical history of benign prostate hypertrophy that was managed using intermittent self-catheterization due to benign prostate hypertrophy was admitted to our hospital with fever, right scrotal pain, and swelling that had manifested 4 weeks prior to admission. He was diagnosed with pyogenic epididymoorchitis and was treated with ceftriaxone 1 g intravenously every 24 h. Urine culture was negative and scrotal ultrasound revealed a hyperechoic testis. Two weeks prior to admission, a follow-up scrotal ultrasound had shown a hypervascularity of the testis and epididymis with hydrocele but no findings of testicular torsion. Since his clinical symptoms had gradually improved, we switched to oral cefpodoxime 200 mg every 12 h. However, as he developed a scrotal abscess and his scrotal skin was torn, he was admitted to our hospital for further treatment. On admission, vital signs were as follows: clear consciousness; temperature, 36.0 °C; respiratory rate, 16 breaths per min; blood pressure, 140/78 mmHg; and pulse rate, 67 beats per min. On physical examination, a scrotal ulcer with exudate, swelling, and redness was observed. He did not have any costovertebral angle tenderness or tenderness of the prostate. Laboratory data revealed 7000/μL (neutrophils 54.9%, lymphocytes 5.6%, monocytes 5.0%) of white blood cells and 0.21 mg/dL of C-reactive protein. Urinalysis revealed neither white nor red blood cells. Urinary Gram staining and culture results were negative. Scrotal ultrasonography showed a hypoechoic, avascular lesion (Fig. 1) without testicular torsion. Computed tomography showed fluid correction with contrast effect in the capsule of the right scrotum (Fig. 2) (Fig. 3).

**Fig. 4:**
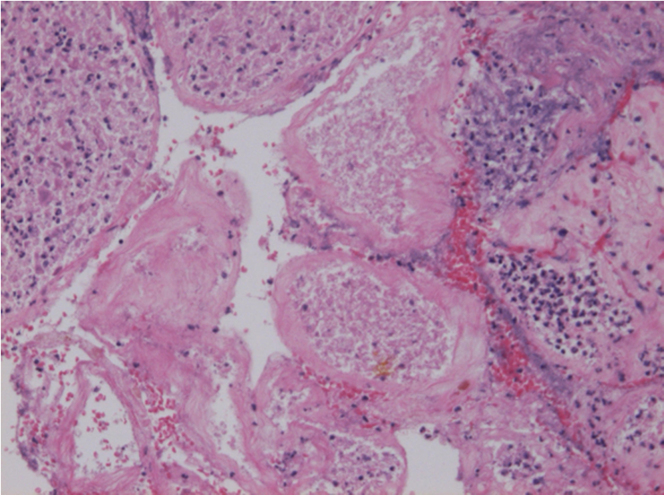
Pathological findings of the specimens (×10 H&E staining). Inflammatory cell infiltration and necrosis in the testicular parenchyma H&E: Hematoxylin and Eosin.

**Fig. 5:**
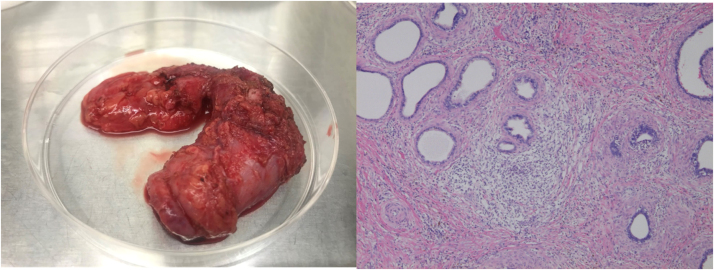
Contents of the scrotum (×4 left: H&E staining). a (left): The spermatic cord, vas deferens, and epididymis were surgically removed, but no testis was found. b (right): The histopathology of epididymis showed subacute inflammatory changes. H&E: Hematoxylin and Eosin.

We changed the antibiotic to cefepime 1 g every 8 h. The abscess in the necrotic tissue was drained by surgical incision, and *Pseudomonas aeruginosa* was detected in the pus culture by matrix-assisted laser desorption ionization-time of flight mass spectrometry (MALDI-TOF Biotyper [Bruker Daltonics, Germany]) and VITEK2 Compact (bioMérieux Inc.). Pathological examination showed inflammatory cell infiltration and necrosis in the testicular parenchyma (Fig. 4). The minimum inhibitory concentrations measured by MicroScan WalkAway 96 Plus and NC-NF2J panel (Beckman Coulter Inc.). Based on the susceptibility results of the pus culture (Table 1), the patient was treated with oral ciprofloxacin 400 mg three times a day. The vital signs and subjective symptoms had stabilized, but the necrosis of the testes was extensive, suggesting that functional preservation was not applicable. Because of the necrosis, his scrotal contents were surgically removed under aseptic conditions 2 months after the resolution of the infection. Pathological examination showed no inflammation of testis and epididymis (Fig. 5). Ciprofloxacin was discontinued 1 month postoperatively. He did not have any evidence of recurrence or urological complications at follow-up 6 months after discharge.

**Table 1:** Antimicrobial susceptibility of *Pseudomonas aeruginosa*.

Antimicrobials	MIC (μg/mL)	Susceptibility
Tobramycin	≤ 2	S
Aztreonam	≤ 2	S
Ceftazidime	≤ 2	S
Piperacillin	≤ 8	S
Ciprofloxacin	≤ 0.25	S
Levofloxacin	≤ 0.5	S
Meropenem	≤ 2	S

MIC: Minimum inhibitory concentration

## Discussion

Two authors independently reviewed the titles and abstracts of database records, retrieved full texts for eligibility assessment, and extracted data from these cases. We did a systematic search using the keywords "Testicular Infarction", "Epididymitis", and "Bacterial Infection." We searched the electronic databases PubMed, Embase, and Ichushi from their inception to December 23, 2020 (process described in Appendix A).

Through the database screening of the literature, we found 34 articles describing 60 cases of testicular necrosis due to acute epididymitis (flow_chart.jpg, Appendix B). The clinical characteristics of the 61 cases, including our case, are shown in Appendix B. The median patient age was 41 years. Forty-three patients had unilateral involvement (left: 21 cases, right: 22 cases), and three cases had bilateral testicular necrosis. Of the 15 out of 39 patients with underlying diseases of the urinary system, such as intermittent catheterization and surgical procedure on benign prostate hypertrophy, 1 out of 39 patients had scrotal trauma. The time from the onset of epididymitis to diagnosis of testicular infarction varied from 1 to 9 months. Regarding the diagnosis of testicular infarction, most cases were diagnosed by ultrasound showing decreased blood flow in the testes, combined with pathological findings. Although less commonly performed, magnetic resonance imaging (MRI), radionuclide imaging, or surgical exploration of the testis are alternative diagnostic methods. Notably, 42 out of the 61 cases had urine culture information available: *Escherichia coli,* 22 cases (one case with budding yeast, another case with *Klebsiella pneumoniae*)*; Klebsiella* sp.*,* 3 cases (culture negative, but *Klebsiella oxytoca* polymerase chain reaction negative in one case, extended-spectrum-beta-lactamase producing *Klebsiella* sp. in one case, *Klebsiella pneumoniae* with *Escherichia coli* in one case); *Pseudomonas* sp.*,* 2 cases; and *Serratia marcescens,* one case, and negative in urine culture in 15 cases. Regarding the surgical intervention, 11 cases did not have surgical information available. In addition, we excluded two cases (refusal of surgery in one case, unavailable surgical detail information in the other). Orchiectomy was performed in of 34 out of 48 cases (71%).

The strength of this report is that it provides detailed information on patient characteristics, underlying diseases, time to diagnosis, causative organisms, diagnostic methods, and treatment of testicular infarction, which is a rare but serious complication of epididymitis. The reasons for testicular infarction during treatment of epididymitis have been suggested to be due to increased exudate production owing to inflammation and tissue edema causing testicular compartment syndrome [2], [13]. It has also been suggested that venous congestion and increased susceptibility to thrombus formation by bacterial exotoxins [3] may lead to tissue circulatory insufficiency and hypoxia [2], [13]. Radionucleotide imaging of blood flow in the scrotum has demonstrated reduced blood flow in the testes of patients with severe epididymitis [4]. In general, the most common causative organisms of epididymitis are *Neisseria gonorrhoeae* and *Chlamydia trachomatis* [5], [6] in younger patients, but our review shows that these rarely lead to testicular infarction. However, in middle-aged and older adults, the causative organisms are usually *Escherichia coli* and *Pseudomonas aeruginosa* [7], [8]. In this review, *Escherichia coli* was the most common causative organism of epididymitis leading to testicular infarction, and it is important to consider the possibility of complications of testicular infarction in non-gonococcal and non-chlamydial epididymitis, although it is rare.

Notably, previous studies have shown that less than 30% of urine cultures submitted for presumed etiology of epididymitis are positive [9]. In our review, approximately 53% (8/15) of patients had negative urine cultures but positive tissue cultures, while 13.3% (2/15) of patients presented a discordance between urine cultures and tissue cultures in surgical specimens. Our patient was on intermittent-self catheterization for benign prostate hypertrophy and was referred to our department for acute epididymitis. Although initial urine culture was negative, his condition clinically improved with third-generation cephalosporin treatment. After several days, his symptoms flared up. The urine culture remained negative, but the culture of incisional scrotal drainage was positive for *Pseudomonas aeruginosa*. It is speculated that *Pseudomonas aeruginosa* entered through intermittent-self catheterization, complicated by testicular infarction, which eventually led to tissue necrosis and abscess. Because it is difficult to obtain tissue culture from all patients with epididymitis, it may be necessary to consider the escalation of antimicrobial agents when the clinical course deteriorates rather than relying solely on the results of urine culture. The diagnosis is often based on the ultrasonographic evaluation of blood flow, as in this case, but it is necessary to differentiate between scrotal abscesses and testicular infarction in the hypoechoic areas of the scrotum [12]. When the diagnosis is difficult, contrast-enhanced MRI is used as an adjunct for diagnosis [10], [11], but MRI is difficult to perform in facilities that do not have access to imaging. In addition, as it is generally difficult to differentiate between testicular tumors and testicular infarction[14], gross and pathologic examinations may be performed through surgical exploration of the scrotum. Therefore, the management of epididymitis requires cooperation with a urologist.

In terms of testicular prognosis, 61% of patients with testicular infarction can be treated conservatively [12]. In this review, orchiectomy was performed in 34 out of the 48 patients (71%). Since 3 out of the 48 cases (2.1%) that were treated conservatively had testicular atrophy and decreased blood flow to the testis, the testicles were hard to preserve and testicular removal was preferable. One of the limitations of this study is that the number of reports may not accurately reflect the number of cases of testicular infarction secondary to epididymitis, because there may be cases in which testicular infarction is not accurately diagnosed and patients may recover with conservative treatment. However, as testicular infarction is an important complication of epididymitis, this report may help clinicians to recognize the presence of this disease, perform frequent blood flow assessment, and carefully monitor the response to antimicrobial agents.

In conclusion, we encountered a case of testicular necrosis during the treatment of epididymitis with a negative urine culture and *Pseudomonas aeruginosa* detected in the tissue culture. Clinicians should consider that urine culture is not sensitive to epididymitis, and that, if the clinical course worsens, antimicrobial escalation should be considered, and blood flow should be frequently evaluated to ensure early urologic intervention.

## Authors' contributions

The manuscript was seen and approved by all the authors and is not under consideration elsewhere. All the authors contributed to the work in this report. KI collected clinical data and wrote the initial draft of the manuscript. KI, TN, and FK performed the systematic review of the literature. TM, YU, and NM supervised and edited the manuscript. The author(s) read and approved the final manuscript.

## Funding

This research did not receive any specific grant from funding agencies in the public, commercial, or not-for-profit sectors.

## CRediT authorship contribution statement

Kazuhiro Ishikawa wrote the manuscript. Nobuyoshi Mori supervised writing the manuscript. Yuki Uehara supervised writing the manuscript. Takahiro Matsuo supervised writing the manuscript. Tomoaki Nakamura supervised writing the manuscript. Fujimi Kawai supervised writing the manuscript.

## Ethics approval and consent to participate

Not applicable.

## Consent for publication

Written informed consent was obtained from the patients in this case report. A copy of written consent is available for the journal.
