# Supplementary material for: Testicular infarction as a rare complication of pyogenic epididymoorchitis due to Pseudomonas aeruginosa: A case report and systematic literature review
Source: IDCases. 2021 Aug 25;26:e01258. doi: 10.1016/j.idcr.2021.e01258 (PMC8416638; doi:10.1016/j.idcr.2021.e01258)
Supplement: Supplementary file 1 — Supplementary material [file mmc1.docx]

Appendix A. Keywords to search the case report of testicular infarction due to epididymitis

PubMed

(("Infarction"[MeSH Terms:noexp] OR "infarct*"[Title/Abstract] OR "loss"[Title/Abstract] OR ("Necrosis"[MeSH Terms:noexp] OR "Necrosis"[Title/Abstract])) AND ("Testicular Diseases"[MeSH Terms:noexp] OR "Testis"[MeSH Terms:noexp] OR ("Testis"[Title/Abstract] OR "testicular"[Title/Abstract])) AND ("Epididymitis"[Text Word] OR ("epididymoorchitis"[Title/Abstract] OR "epididymo-orchitis"[Title/Abstract] OR "orchiepididymitis"[Title/Abstract]))) OR (("Infarction"[MeSH Terms:noexp] OR "infarct*"[Title/Abstract] OR "loss"[Title/Abstract] OR ("Necrosis"[MeSH Terms:noexp] OR "Necrosis"[Title/Abstract])) AND ("Testicular Diseases"[MeSH Terms:noexp] OR "Testis"[MeSH Terms:noexp] OR ("Testis"[Title/Abstract] OR "testicular"[Title/Abstract])) AND ("Bacterial Infections"[MeSH Terms] OR "bacteria*"[Text Word] OR "Bacteria"[MeSH Terms]))

Embase

(((((('infarction'/de OR 'infarction') OR infarct* OR loss OR ('necrosis'/de OR 'necrosis')) AND (('testis disease'/de OR 'testis disease') OR ('testis'/de OR 'testis') OR testicular)) AND (('epididymitis'/exp OR 'epididymitis') OR (epididymoorchitis OR 'epididymo orchitis' OR orchiepididymitis OR epididymitides))) OR (((('infarction'/de OR 'infarction') OR infarct* OR loss OR ('necrosis'/de OR 'necrosis')) AND (('testis disease'/de OR 'testis disease') OR ('testis'/de OR 'testis') OR testicular)) AND ('bacterial infection'/exp OR 'bacterium'/exp OR bacteri*))) AND [embase]/lim) NOT (((((('infarction'/de OR 'infarction') OR infarct* OR loss OR ('necrosis'/de OR 'necrosis')) AND (('testis disease'/de OR 'testis disease') OR ('testis'/de OR 'testis') OR testicular)) AND (('epididymitis'/exp OR 'epididymitis') OR (epididymoorchitis OR 'epididymo orchitis' OR orchiepididymitis OR epididymitides))) OR (((('infarction'/de OR 'infarction') OR infarct* OR loss OR ('necrosis'/de OR 'necrosis')) AND (('testis disease'/de OR 'testis disease') OR ('testis'/de OR 'testis') OR testicular)) AND ('bacterial infection'/exp OR 'bacterium'/exp OR bacteri*))) AND [embase]/lim AND ([medline]/lim OR [pubmed-not-medline]/lim))

Ichushi

((((Testis [Japanese]/TH or Testis [Japanese]/TA)) and (((Infarction [Japanese]/TH or Infarction [Japanese]/TA)) or ((Necrosis [Japanese]/TH or Necrosis [Japanese]/TA)) or (Loss [Japanese]/TA))) and ((Epididymitis [Japanese]/TH or Epididymis [Japanese]/AL))) or ((((Testis [Japanese]/TH or Testis [Japanese]/TA)) and (((Infarction [Japanese]/TH or Infarction [Japanese]/TA)) or ((Necrosis [Japanese]/TH or Necrosis [Japanese]/TA)) or (Loss [Japanese]/TA))) and ((Bacterial infection [Japanese]/TH) or (Bacteria [Japanese]/TH) or (Bacteria [Japanese]/TA or Infection[Japanese]/TA or Bacteria [Japanese]/TA))).
