# Supplementary material for: Testicular infarction as a rare complication of pyogenic epididymoorchitis due to Pseudomonas aeruginosa: A case report and systematic literature review
Source: IDCases. 2021 Aug 25;26:e01258. doi: 10.1016/j.idcr.2021.e01258 (PMC8416638; doi:10.1016/j.idcr.2021.e01258)
Supplement: Supplementary file 2 — Supplementary material [file mmc2.docx]

Appendix B. Clinical characteristics of cases of testicular infarction due to pyogenic epididymoorchitis

| Patient number | Case reference | Age (years) | Published year | Published country | Underlying diseases | Initial chief complaint | Laterality | Chief complaint due to testicular infarction | Duration from onset to testicular necrosis | Urine culture | Diagnostic modality of testicular infarction | Hydrocele | Histological culture | Antibiotics sensitivity | Histopathological diagnosis of testicular infarction | Antimicrobial used/duration | Surgical intervention/clinical outcome | Complications |
| --- | --- | --- | --- | --- | --- | --- | --- | --- | --- | --- | --- | --- | --- | --- | --- | --- | --- | --- |
| 1[1] | Ishikawa T. | 21 | 2017 | Japan | Mild developmental disorder | Scrotal pain | Right | Palpable right testicular nodule | 11 weeks | N/A | MRI | No | N/A | N/A | Yes | LVFX 2 months ago | Orchiectomy | N/A |
| 2[2] | Lee W. | 37 | 2016 | Korea | None | Fever, severely painful left scrotal swelling | Left | Recurring initial symptoms | 13 days | Initial culture-negative, but *K. oxytoca* PCR-positive at the testicular infarction | US | No | *K. oxytoca*  PCR-positive | N/A | Yes | CBPZ 2 g/day for 11 days, TFLX 450 mg/day for 2 days | Orchiectomy | None |
| 3[3] | Ibrahimi A. | 23 | 2020 | Morocco | None | N/A | Right | Worsening right testicular pain | 11 days | N/A | US | No | N/A | N/A | Yes | N/A | Orchiectomy | None |
| 4[4] | Parkin C.J. | 72 | 2020 | Australia | *Aspergillus* pneumonia, cavernous sinus thrombosis, prostate cancer | N/A | Left | 2-week history of worsening pain and swelling of his left testis | 16 days | *E. coli* | US/SE | Yes | N/A | CEX(S), AMPC(R) | Yes | CEX → IV antibiotics (N/A) | Non-viable testicular tissue debridement | N/A |
| 5[5] | Ramjit A. | 41 | 2020 | USA | N/A | No prior episode of epididymitis | Left | Sudden-onset left groin pain | 11 days | *E. coli* | US/MRI | Yes | Negative | N/A | N/A | CTRX for 4 days → CPFX+MNZ | Refused orchiectomy | N/A |
| 6[6] | Ong Lay Keat W. | 49 | 2020 | Malaysia | HBV, HTN | Fever, scrotal pain and swelling | Bilateral | Initial symptoms persistent | 26 days | Negative | US | No | *E. coli* | N/A | Yes | Oral A/S for 1 week → IV A/C → IV CPFX 400 mg every 12 hours → CXM | Bilateral orchiectomy | None |
| 7[7] | Alharbi B. | 18 | 2019 | Saudi Arabia | None | Scrotal pain and swelling for 2 days | Left | Unresolved symptoms and experiencing chills and fever | 4 days | N/A | USI | Yes | *E. coli* | N/A | Yes | Oral CPFX + DOXY → P/T 4.5 g every 8 hours for 4 days → oral CFPX 400 mg every 12 hours for 2 weeks | Orchiectomy | None |
| 8[8] | Matsumoto E. | 69 | 2017 | USA | HTN,DM | Scrotal pain for 3 days | Left | Persistent left scrotal pain 8 days after the initial visit | 11 days | *E. coli* | US | Yes | N/A | ST(S) | N/A | ST | Orchiectomy | None |
| 9[9] | D. Chia | 50 | 2016 | Australia | None | Macroscopic hematuria, testicular pain and dysuria for 4 days | Left | Worsening scrotal swelling and pain | 23 days | *E. coli* | US | No | N/A | N/A | Yes | CEX → CTRX + GM for 48 hours → NFLX for 2 weeks → MFIPC + GM → CPFX for 4 weeks | Orchiectomy | None |
| 10[10] | Fehily S.R. | 49 | 2015 | Australia | HIV | Severe testicular pain and swelling associated with dysuria and macroscopic hematuria | Bilateral | 2 months later with recurrent left-sided epididymoorchitis → 7 months later, presented with right-sided epididymoorchitis | 9 months | *E. coli* | US | No | N/A | GM(S), CEX(S), NFLX(S), A/C(S) | Yes | ST → CEX 500 mg twice daily → CTRX for 3 days → CPFX for 6 weeks, on right side exacerbation, CTRX 1 g/day for 7 days → CPFX 750 mg every 12 hours for 6 weeks | Right orchiectomy/left sided cured | None |
| 11[10] | Fehily S.R. | 27 | 2015 | Australia | Recurrent urinary tract infections, *Chlamydia trachomatis* epididymoorchitis | Symptoms of epididymoorchitis | Left | While still on ciprofloxacin, the patient developed recurrent left-sided epididymoorchitis | 6–12 weeks | Negative | US | No | *K. pneumoniae* | CPFX(S) | Yes | NFLX → single dose of GM 400 mg + CTRX 1 g/day for 4 days → single dose of oral AZM 1 g + NFLX 400 mg twice a day for 6 weeks → IV CPFX 400 mg every 8 hours → oral CPFX 500 mg twice a day for 6 weeks combination of DOXY 100 mg twice a day for 2 weeks | Orchiectomy | NA |
| 12[11] | Farber N.J. | 58 | 2013 | USA | DM, TRUS-guided prostate biopsy | Hemi-scrotum pain | Right | The pain progressed over the next several days without associated fever or chills | 2 weeks | *E. coli* | US | Yes | N/A | LVFX(R), ABPC(R), ST(R), DOXY(R) | N/A | Oral CPFX → IM CTRX → oral DOXY → IV LVFX → ABPC + GM → CXM → CTRX for 4 weeks | Orchiectomy | none |
| 13[12] | Chin S.C. | 42 | 1998 | Taiwan | None | Painful scrotal swelling for 2 days | Right | N/A | 4 days | Negative | US | No | N/A | N/A | Yes | N/A | orchiectomy | N/A |
| 14[13] | Eisner D.J. | 65 | 1991 | USA | TURP due to BPH | Scrotal pain and swelling | Bilateral | The scrotum and epididymal area remained swollen | 2 weeks | *P. aeruginosa* | US/RI | No | N/A | GM(R) | Yes | ABPC + GM → oral CPFX + IV TOB | right orchiectomy/ left sided cured | N/A |
| 15[14] | Yusuf G. | 55 | 2013 | UK | Resected colorectal cancer | Vague lower urinary tract symptoms for 3 days | Right | Increased pain in the right hemi-scrotum and suprapubic region | About 3 days | *E. coli* | US | No | N/A | N/A | Yes | Antibiotics (N/A) | Orchiectomy | None |
| 16[14] | Yusuf G. | 64 | 2013 | UK | DM, IHD, HTN | Hemi-scrotal swelling and pain of 2 days | Left | Persisting pain for 2 more days | N/A | N/A | US | Yes | N/A | N/A | Yes | Antibiotics (N/A) | Partial orchiectomy | None |
| 17[15] | Sue S.R. | 43 | 1998 | USA | HCV, unprotected sexual intercourse 3 weeks ago | Groin and testicular pain radiating to his lower abdomen for 2 days | Left | Persistent scrotal pain | About 3 weeks | Negative | US | No | N/A | N/A | Yes | Oral quinolone → CTRX + GM for 2 days → oral quinolone + DOXY | Orchiectomy | N/A |
| 18[16] | Marks R. | 50 | 2009 | USA | None | Testicular pain | Right | Continued testicular pain | 6 days | *E. coli* | US | Yes | *C. albicans* | N/A | Yes | Antibiotics (N/A), antifungals (N/A) | Orchiectomy | None |
| 19[17] | Hackett B. | 67 | 2020 | USA | HTN, CKD, intermittent catheterization due to BPH | Scrotal pain and swelling for a week, chills, fever, nausea, and vomiting pain radiating to the inguinal region | Right | Persistent febrile, interval worsening of scrotal edema | 10 days | *K. pneumoniae*, *E. coli* | SE | Yes | *S. lugdunensis* | N/A | Yes | P/T → CEX | Orchiectomy | None |
| 20[18] | Devlies W. | 35 | 2020 | Belgium | None | Swelling, fever, and hemi-scrotum pain for a week | Right | Symptomatology did not improve notably | 9 days | *E. coli* | US | Yes | N/A | CTRX(S), temocillin(S), fluoroquinolones(R) | N/A | CPFX 500 mg → CTRX 2 g/day + AMK 1 g/day → temocillin for 7 days | Orchiectomy | None |
| 21[19] | Wang X. | 29 | 2020 | China | Left varicocele, hypokalemia | Fever and severely painful scrotal swelling for 3 days | Right | Right scrotal swelling and pain recurred | 38 days | *E. coli* | US | No | *E. coli* | N/A | Not infarction, but inflammatory infiltration within the seminiferous tubules and interstitial with multiple micro-abscesses | Antibiotics (N/A) | Orchiectomy | None |
| 22[19] | Wang X. | 36 | 2020 | China | Hepatic cyst, gallbladder polyps | Scrotal dull pain for a month | Right | Severe pain and scrotal swelling for 7 days | 49 days | Negative | US | Yes | Negative | N/A | Yes | Antibiotics (N/A) | Orchiectomy | None |
| 23[20] | Rhudd A. | 37 | 2017 | UK | None | Testicular pain for 3 days | Left | Worsening of his pain | About 4 days | *E. coli* | US | No | N/A | N/A | Yes | Oral CPFX | Orchiectomy | N/A |
| 24[21] | Smets T. | 36 | 2017 | Belgium | None | Severe scrotal pain | Left | Aggravated testicular pain and swelling despite painkillers and anti-inflammatory medication | 1 month | N/A | US/SE | No | N/A | None | N/A | N/A | Bilateral orchidopexy/testicle spared/remaining hypo-echogenicity | None |
| 25[22] | Suciu M. | 47 | 2017 | Romania | N/A | Intense nocturnal pain and the swelling of the scrotum | Left | Edema and moderate swelling of the left side of the scrotum, accompanied by dysuria, fever from a month ago | A month | ESBL *Klebsiella* sp. | US | No | N/A | N/A | Yes | CPFX for 1 month | Orchiectomy | N/A |
| 26[23] | Parambath A.N. | 31 | 2010 | Qatar | N/A | Acute scrotal pain, swelling, dysuria, and fever | Right | Increased scrotal pain and swelling | 3 days from initial visiting | N/A | US | No | N/A | N/A | Yes | Antibiotics (N/A) | Orchiectomy | N/A |
| 27[24] | Gerscovich E.O. | 41 | 2008 | USA | Spina bifida, neurogenic bladder, intermittent catheterization for 2 years | Gradual enlargement of the left scrotum and increasing flank and groin pain for 2 days | Left | A lack of resolution of the clinical symptoms | About 3 weeks | *S. marcescens* | US | No | N/A | N/A | Yes | CTRX for 3 days → oral antibiotics (N/A) for 10 days | Orchiectomy | N/A |
| 28[25] | Natarajan V. | 47 | 1995 | UK | Lower UTI due to *E. coli*, DM | Painful swelling of the testis for 1 week | Left | Persistent testicular tenderness | 9 days | Negative | US/SE | No | *E. coli* | Antibiotics (S) | Yes | Antibiotics (N/A) | Orchiectomy | None |
| 29[26]] | Sanders L.M. | 54 | 1994 | New Zealand | Quadriplegic | N/A | Left | Left testicular pain, swelling, scrotal erythema, and fever | N/A | N/A | US/RI | No | GPC | N/A | Yes | ABPC + AMK for 5 days → CEZ + GM | Surgery (unknown) | N/A |
| 30[27] | Rencken R.K. | 55 | 1990 | South Africa | TUR-BT for superficial transitional-cell carcinoma, removed cystoscopically for superficial grade Ⅱ transitional-cell carcinoma | Scrotal pain and swelling for 3 days | Right | Although the pain began to subside due to antibiotics, but the induration of the right cord, epididymis and testis remained | N/A | N/A | US/RI | No | N/A | N/A | Yes | TOB 80 mg every 8 hours + ABPC 500 mg every 6 hours | Orchiectomy | N/A |
| 31[28] | Owen E.R. | 31 | 1990 | UK | A week’s history of a painful left-sided scrotal swelling | None | Left | Remains a tender swelling in the scrotum | 4 weeks | N/A | US/SE | No | N/A | N/A | Yes | Antibiotics (N/A) | Orchiectomy | N/A |
| 32[29] | Vieras F. | 13 | 1986 | USA | Kicked in the groin while playing football | Testicular pain and swelling for a day | Left | The scrotum looked better but the firmness and hardness had not changed | About 2 weeks | N/A | RI | N/A | N/A | N/A | Yes | Antibiotics (N/A) | Orchiectomy | N/A |
| 33[30] | Bird K. | 22 | 1984 | USA | N/A | Pain and swelling of the testis for 10 days | Right | A painful mass 1.5 cm in diameter in the lower pole of the right testicle, with minimal skin thickening and induration over the lower right hemiscrotum | 10 days | N/A | SE | None | N/A | N/A | Yes | TC | Orchiectomy | N/A |
| 34[30] | Bird K. | 17 | 1984 | USA | N/A | A painful right scrotum | Right | The testicle remained hard and firm, only becoming soft 1 week before admission. | 6 weeks | Negative | SE | None | N/A | N/A | Yes | Antibiotics (N/A) | Orchiectomy | N/A |
| 35[30] | Bird K. | 56 | 1984 | USA | Prostatitis 2 weeks PTA | None | Left | Fever and severe left scrotal pain | 2 weeks | *E. coli*, budding yeast | RI | Yes | N/A | N/A | Yes | ABPC | Orchiectomy | N/A |
| 36[30] | Bird K. | 79 | 1984 | USA | Prostatectomy 2 years before, kidney stones | None | Bilateral | Increasing scrotal edema and tenderness for 6 days prior to admission, with chills and fever commencing 3 days later. | 6 days | *E. coli* | US | Yes | N/A | N/A | Yes | N/A | Orchiectomy | N/A |
| 37[31] | Vordermark J.S. 2nd | 22 | 1982 | USA | N/A | Moderately severe epididymitis | N/A | Epididymitis progressed after 24 hours of medical therapy and the patient began having fevers to 103°F | 1 day | Negative | RI | No | N/A | N/A | N/A | CEX | Orchiectomy | None |
| 38[31] | Vordermark J.S. 2nd | 25 | 1982 | USA | N/A | Severe epididymitis and scrotal edema | N/A | The condition was unchanged after 5 days of therapy | 5 days | Negative | RI | No | N/A | N/A | N/A | DOXY | Orchiectomy | N/A |
| 39[31] | Vordermark J.S. 2nd | 21 | 1982 | USA | N/A | Second episode of epididymitis associated with an *E.coli* UTI | N/A | The temperature spiked to 102°F and the patient complained of a marked increase in pain in the involved testicle | N/A | *E. coli* | RI | No | N/A | Antibiotics (S) | N/A | CEX | Epididymectomy/clinical improvement in perfusion to the testicle |  |
| 40[32] | Kirk D. | 21 | 1982 | UK | N/A | None | Left | 6-week history of pain and swelling of his left testis | 6 weeks | N/A | SE | No | N/A | N/A | Yes | N/A | Orchiectomy | N/A |
| 41[32] | Kirk D. | 36 | 1982 | UK | N/A | Pain and swelling of the scrotum | Right | The body of the testis was hard and clinically indistinguishable from a tumor | 2 months | N/A | SE | No | N/A | N/A | Yes | ABPC | Orchiectomy | N/A |
| 42[33] | Costas S. | 26 | 1973 | South Africa | N/A | Pain in the testis | Left | Mildly pyrexial, the pain increased | 2 days | N/A | SE | Yes | N/A | N/A | N/A | ABPC 500 mg every 6 hours | Epididymectomy/testicular atrophy | None |
| 43[33] | Costas S. | 16 | 1973 | South Africa | N/A | Pain in the scrotum | Left | A slightly tender, conglomerate mass in the scrotum | N/A | Negative | SE | N/A | N/A | N/A | N/A | PCG+SM for 3 days → GM for 9 days → oral ABPC | Orchiectomy | N/A |
| 44[33] | Costas S. | 20 | 1973 | South Africa | Gun-shot wound of the bladder and rectum resulting in a rectovesical fistula. sigmoid colostomy, prolonged catheter drainage | None | N/A | Acute epididymitis which did not settle on conservative therapy | N/A | *Pseudomonas* sp. | SE | N/A | N/A | N/A | N/A | N/A | Epididymectomy/testicular atrophy | None |
| 45[33] | Costas S. | 32 | 1973 | South Africa | Fracture of the pelvis | Swollen, tender painful testis for 6 days | Right | Local signs had deteriorated with tenderness and swelling of the cord up to the external inguinal ring | 7 days | *E. coli* | SE | N/A | Negative | N/A | N/A | N/A | Epididymectomy/N/A | N/A |
| 46[33] | Costas S. | 37 | 1973 | South Africa | N/A | Gradually increasing pain and swelling of the testis for 5 days | Right | Deterioration of the local signs | 6 days | Negative | SE | N/A | N/A | N/A | N/A | AMPC → GM 80 mg every 8 hours | No epididymectomy, testicular normal incision of the external inguinal ring due to the constriction of the ring | None |
| 47[33] | Costas S. | 36 | 1973 | South Africa | N/A | Pain in the testis and urethral discharge for 7 days | Right | More pain associated with increased tenderness and swelling of the cord | 8 days | N/A | SE | Yes | N/A | N/A | N/A | ABPC | Incision of the external inguinal ring but not available of testicular findings due to lost follow-up | N/A |
| 48[33] | Costas S. | 23 | 1973 | South Africa | N/A | Painful testis and urethral discharge for 4 days | Right | Deterioration of the local signs and symptoms | 6 days | N/A | SE | Yes | N/A | N/A | N/A | ABPC | Incision of the external inguinal ring | None |
| 49[34] | Hourihane D.O. | 55 | 1970 | Ireland | N/A | N/A | N/A | N/A | N/A | N/A | N/A | N/A | *E. coli* | N/A | N/A | N/A | N/A | N/A |
| 50[34] | Hourihane D.O. | 43 | 1970 | Ireland | N/A | N/A | N/A | N/A | N/A | *E. coli* | N/A | N/A | N/A | N/A | N/A | N/A | N/A | N/A |
| 51[34] | Hourihane D.O. | 30 | 1970 | Ireland | N/A | N/A | N/A | N/A | N/A | Negative | N/A | N/A | *E. coli* | N/A | N/A | N/A | N/A | N/A |
| 52[34] | Hourihane D.O. | 56 | 1970 | Ireland | N/A | N/A | N/A | N/A | N/A | N/A | N/A | N/A | *E. coli* | N/A | N/A | N/A | N/A | N/A |
| 53[34] | Hourihane D.O. | 30 | 1978 | Ireland | N/A | N/A | N/A | N/A | N/A | *E. coli* | N/A | N/A | *E. coli* | N/A | N/A | N/A | N/A | N/A |
| 54[34] | Hourihane D.O. | 36 | 1970 | Ireland | N/A | N/A | N/A | N/A | N/A | Negative | N/A | N/A | *Proteus* sp. | N/A | N/A | N/A | N/A | N/A |
| 55[34] | Hourihane D.O. | 71 | 1970 | Ireland | N/A | N/A | N/A | N/A | N/A | *E. coli* | N/A | N/A | *E. coli* | N/A | N/A | N/A | N/A | N/A |
| 56[34] | Hourihane D.O. | 48 | 1970 | Ireland | N/A | N/A | N/A | N/A | N/A | Negative | N/A | N/A | *E. coli* | N/A | N/A | N/A | N/A | N/A |
| 57[34] | Hourihane D.O. | 42 | 1970 | Ireland | N/A | N/A | N/A | N/A | N/A | Negative | N/A | N/A | *E. coli* | N/A | N/A | N/A | N/A | N/A |
| 58[34] | Hourihane D.O. | 45 | 1970 | Ireland | N/A | N/A | N/A | N/A | N/A | *E. coli* | N/A | N/A | N/A | N/A | N/A | N/A | N/A | N/A |
| 59[34] | Hourihane D.O. | 57 | 1970 | Ireland | N/A | N/A | N/A | N/A | N/A | N/A | N/A | N/A | *E. coli* | N/A | N/A | N/A | N/A | N/A |
| 60[34] | Hourihane D.O. | 74 | 1970 | Ireland | Hernia repair, retropubic prostatectomy | N/A | N/A | N/A | N/A | *E. coli* | N/A | N/A | *E. coli* | N/A | N/A | N/A | N/A | N/A |
| 61 | K. Ishikawa | 64 | 2021 | Japan | Intermittent catheterization due to BPH | Fever, scrotal pain, and swelling | Right | A scrotal ulcer with exudate, swelling, and redness | 4 weeks | Negative | US | Yes | *P. aeruginosa* | N/A | Yes | CTRX → CPDX → CFPM → CPFX for a total of about 4 months | Orchiectomy | None |

ABPC: Ampicillin, A/C: Amoxicillin/clavulanate, AMK: Amikacin AMPC: Amoxicillin, A/S: Ampicillin/sulbactam, AZM: Azithromycin, BPH: Benign prostate hypertrophy, *C. albicans*: *Candida albicans*, CBPZ: Cefbuperazone, CEZ: Cefazolin, CEX: Cefalexin, CKD: Chronic kidney disease, CTRX: Ceftriaxone, CPFX: Ciprofloxacin, CXM: Cefuroxime, CPDX: Cefpodoxime, DM: Diabetes mellitus, DOXY: Doxycycline, *E.coli*: *Escherichia coli,* ESBL: Extended-spectrum β-lactamase, GM: Gentamicin, GPC: Gram positive cocci, HBV: Hepatitis B virus, HCV: Hepatitis C virus, HIV: Human immunodeficiency virus, HTN: Hypertension, IHD: Ischemic heart disease, IM: Intramuscular, IV: Intravenous, *K. oxytoca*: *Klebsiella oxytoca,* *K. pneumoniae*: *Klebsiella pneumoniae,* LVFX: Levofloxacin, MFIPC: Flucloxacillin, MNZ: Metronidazole, MRI: Magnetic resonance imaging, N/A: Not applicable, NFLX: Norfloxacin, *P. aeruginosa*: *Pseudomonas aeruginosa*, PCG: Penicillin G, PCR: Polymerase chain reaction, P/T: Piperacillin/tazobactam, R: Resistant, RI: Radioisotope, SE: Surgical exploration, SM: Streptomycin, *S. marcescens: Serratia marcescens*, ST: sulfamethoxazole-trimethoprim, TC: Tetracycline, TFLX: Tosufloxacin, TOB: Tobramycin, TUR-BT: Transurethral resection of the bladder tumor, TRUS: Transurethral resection, TURP: Transurethral resection of the prostate, US: Ultrasound, UTI: Urinary tract infection

1. Ishikawa T, Izumi K, Kondo T, Miyama K, Osada Y, Hayashi H, et al. [Segmental testicular infarction in a patient with epididymitis]. Hinyokika Kiyo 2017;63:387–90. https://doi.org/10.14989/ActaUrolJap_63_9_387.
2. Lee W, Park H, Lee G. A case of testicular infarction from the complications of Klebsiella oxytoca induced acute epididymitis. J Infect Chemother 2016;22:254–6. https://doi.org/10.1016/j.jiac.2015.09.011.
3. Ibrahimi A, Ziani I, Bellouki O, El Sayegh H, Benslimane L, Nouini Y. Epididymo-testicular ischemia without torsion. Urol Case Rep 2020;33:101324. https://doi.org/10.1016/j.eucr.2020.101324.
4. Parkin CJ, Kam J, Yuminaga Y, Winter M. Segmental testicular infarction, a rare complication of epididymo-orchitis. Urol Case Rep 2020;32:101246. https://doi.org/10.1016/j.eucr.2020.101246.
5. Ramjit A, Shin C, Hayim M. Complete testicular infarction secondary to epididymoorchitis and pyocele. Radiol Case Rep 2020;15:420–3. https://doi.org/10.1016/j.radcr.2020.01.001.
6. Ong Lay Keat W, Lechmiannandan S, Manoharan D, Lee SB, Nagalingam P. Case report of bilateral testicular infarction due to severe bilateral epididymo-orchitis: A catastrophic complication causing castration. Int J Surg Case Rep 2020;73:161–3. https://doi.org/10.1016/j.ijscr.2020.07.013.
7. Alharbi B, Rajih E, Adeoye A, Allatiefe BA, Abdullah MH. Testicular ischemia secondary to epididymo-orchitis: A case report. Urol Case Rep 2019;27:100893. https://doi.org/10.1016/j.eucr.2019.100893.
8. Matsumoto E. et al: Consultant (2017) 57:4. Date of Publication: 1 Apr 2017
9. Chia D, Penkoff P, Stanowski M, Beattie K, Wang AC. Testicular infarction and rupture: an uncommon complication of epididymo-orchitis. J Surg Case Rep 2016;2016:rjw077. https://doi.org/10.1093/jscr/rjw077.
10. Fehily SR, Trubiano JA, McLean C, Teoh BW, Grummet JP, Cherry CL, et al. Testicular loss following bacterial epididymo-orchitis: Case report and literature review. Can Urol Assoc J 2015;9:E148–51. https://doi.org/10.5489/cuaj.2174.
11. Farber NJ, Slater RC, Maranchie JK. Multidrug resistant epididymitis progressing to testicular infarct and orchiectomy. Case Rep Urol 2013;2013:645787. https://doi.org/10.1155/2013/645787.
12. Chin SC, Wu CJ, Chen A, Hsiao HS. Segmental hemorrhagic infarction of testis associated with epididymitis. J Clin Ultrasound 1998;26:326–8. https://doi.org/10.1002/(sici)1097-0096(199807/08)26:6<326::aid-jcu10>3.0.co;2-0.
13. Eisner DJ, Goldman SM, Petronis J, Millmond SH. Bilateral testicular infarction caused by epididymitis. AJR Am J Roentgenol 1991;157:517–9. https://doi.org/10.2214/ajr.157.3.1872237.
14. Yusuf G, Sellars ME, Kooiman GG, Diaz-Cano S, Sidhu PS. Global testicular infarction in the presence of epididymitis: clinical features, appearances on grayscale, color Doppler, and contrast-enhanced sonography, and histologic correlation. J Ultrasound Med 2013;32:175–80. https://doi.org/10.7863/jum.2013.32.1.175.
15. Sue SR, Pelucio M, Gibbs M. Testicular infarction in a patient with epididymitis. Acad Emerg Med 1998;5:1128–30. https://doi.org/10.1111/j.1553-2712.1998.tb02679.x.
16. Marks R, McNeil K. Significance of reversal of diastolic blood flow in the evolution of testicular infarction as a complication of epididymo-orchitis. J Radiol Case Rep 2009;3:21–5. https://doi.org/10.3941/jrcr.v3i6.218.
17. Hackett B, Sletten Z, Bridwell RE. Testicular abscess and ischemia secondary to epididymo-orchitis. Cureus 2020;12:e8991. https://doi.org/10.7759/cureus.8991.
18. Devlies W, Seghers M, Dilen K. Case report on secondary testicular necrosis due to fulminant epididymitis: ultrasonographic evaluation and diagnosis. BMC Urol 2020;20:115. https://doi.org/10.1186/s12894-020-00655-w.
19. Wang X, Zhang Z, Fang LK, Chen D, Peng N, Thakker PU, et al. Challenges in the diagnosis of testicular infarction in the presence of prolonged epididymitis: three cases report and literature review. J Xray Sci Technol 2020;28:809–19. https://doi.org/10.3233/XST-200671.
20. Rhudd A, Moghul M, Reid G. Epididymo-orchitis causing testicular infarction: a serious complication of a common disorder. J Surg Case Rep 2017;2017:rjx207. https://doi.org/10.1093/jscr/rjx207.
21. Smets T, Reichman G, Michielsen D. Segmental testicular infarction: a case report. J Med Case Rep 2017;11:140. https://doi.org/10.1186/s13256-017-1308-1.
22. Suciu M, Serban O, Iacob G, Lucan C, Badea R. Severe acute epididymo-orchitis complicated with abscess and testicular necrosis - case report. Ultrasound Int Open 2017;3:E45–7. https://doi.org/10.1055/s-0042-122149.
23. Parambath AN, Omar AJ, Al Hilili SA, Darwish A. Segmental infarction of testis: A rare complication of acute epididymitis. Oman Med J 2010;25:e015. https://doi.org/10.5001/omj.2010.95.
24. Gerscovich EO, Bateni CP, Kazemaini MR, Gillen MA, Visis T. Reversal of diastolic blood flow in the testis of a patient with impending infarction due to epididymitis. J Ultrasound Med 2008;27:1643–6. https://doi.org/10.7863/jum.2008.27.11.1643.
25. Natarajan V, Burgess NA, Gaches CG, Ball RY. Emphysematous infarction of the testis following epididymo-orchitis. Br J Urol 1995;76:270–1. https://doi.org/10.1111/j.1464-410x.1995.tb07696.x.
26. Sanders LM, Haber S, Dembner A, Aquino A. Significance of reversal of diastolic flow in the acute scrotum. J Ultrasound Med 1994;13:137–9. https://doi.org/10.7863/jum.1994.13.2.137.
27. Rencken RK, du Plessis DJ, de Haas LS. Venous infarction of the testis--a cause of non-response to conservative therapy in epididymo-orchitis. A case report. S Afr Med J 1990;78:337–8.
28. Owen ER, Kitson JL, Green B. Venous infarction of the testis secondary to acute epididymitis. Br J Urol 1990;65:107–8. https://doi.org/10.1111/j.1464-410x.1990.tb14672.x.
29. Vieras F. Evolution of acute epididymitis to testicular infarction. Scintigraphic demonstration. Clin Nucl Med 1986;11:158–60. https://doi.org/10.1097/00003072-198603000-00004.
30. Bird K, Rosenfield AT. Testicular infarction secondary to acute inflammatory disease: demonstration by B-scan ultrasound. Radiology 1984;152:785–8. https://doi.org/10.1148/radiology.152.3.6463261.
31. Vordermark JS 2nd, Favila MQ. Testicular necrosis: a preventable complication of epididymitis. J Urol 1982;128:1322–4. https://doi.org/10.1016/s0022-5347(17)53482-5.
32. Kirk D, Gingell JC, Feneley RC. Infarction of the testis: a complication of epididymitis. Br J Urol 1982;54:311–2. https://doi.org/10.1111/j.1464-410x.1982.tb06985.x.
33. Costas S, van Blerk PJ. Incision of the external inguinal ring in acute epididymitis. Br J Urol 1973;45:555–8. https://doi.org/10.1111/j.1464-410x.1973.tb06821.x.
34. Hourihane DO. Infected infarcts of the testis: a study of 18 cases preceded by pyogenic epididymoorchitis. J Clin Pathol 1970;23:668–75. https://doi.org/10.1136/jcp.23.8.668.
