# Supplementary figures and images for: Testicular infarction as a rare complication of pyogenic epididymoorchitis due to Pseudomonas aeruginosa: A case report and systematic literature review
Source: IDCases. 2021 Aug 25;26:e01258. doi: 10.1016/j.idcr.2021.e01258 (PMC8416638; doi:10.1016/j.idcr.2021.e01258)

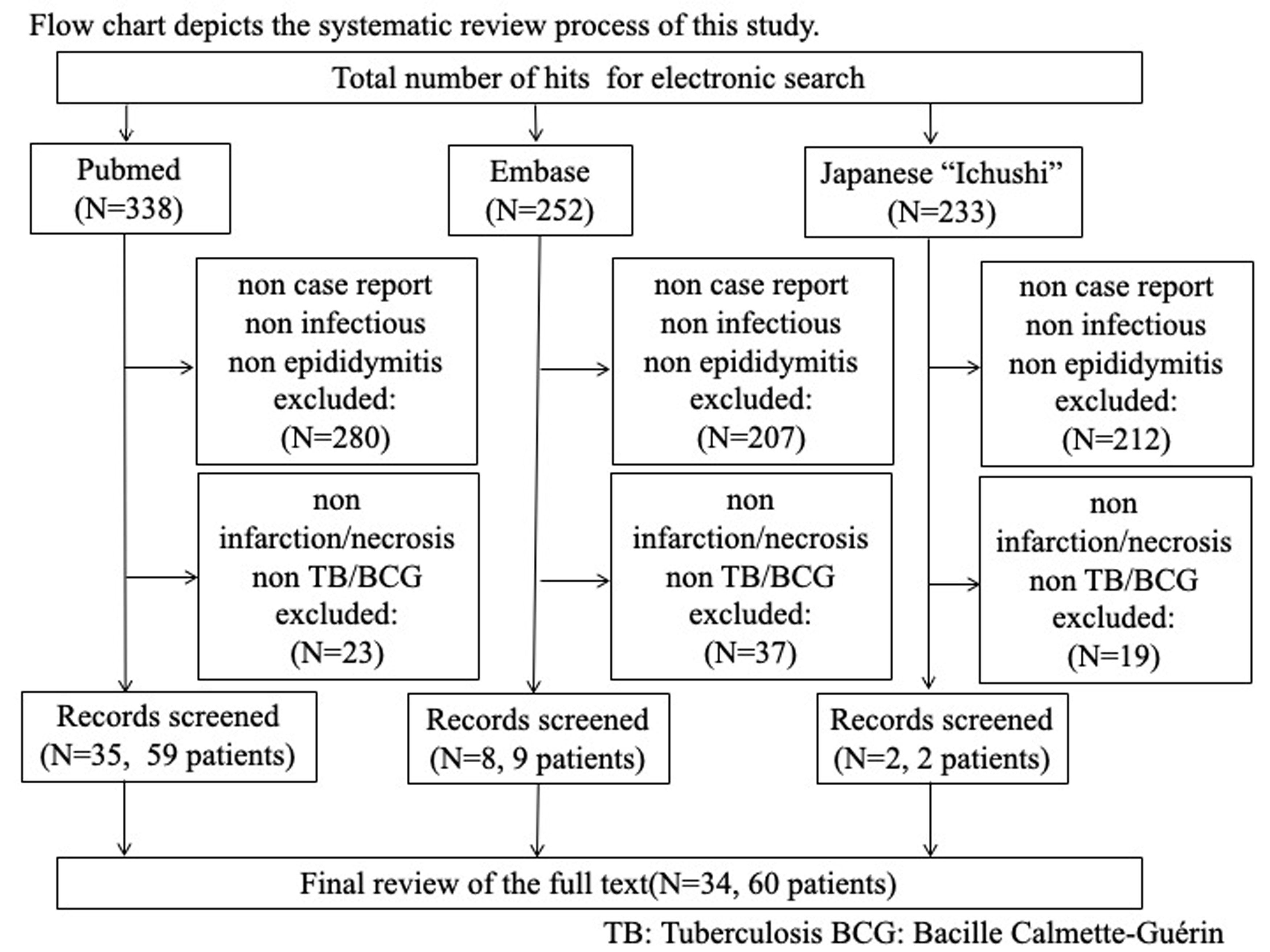

Supplement: Supplementary file 3 — Supplementary material [file mmc3.jpg]
